# The effectiveness of long-lasting, insecticide-treated nets in a setting of pyrethroid resistance: a case–control study among febrile children 6 to 59 months of age in Machinga District, Malawi

**DOI:** 10.1186/s12936-015-0961-3

**Published:** 2015-11-17

**Authors:** Don P. Mathanga, Dyson A. Mwandama, Andy Bauleni, Joseph Chisaka, Monica P. Shah, Keren Z. Landman, Kim A. Lindblade, Laura C. Steinhardt

**Affiliations:** Malaria Alert Centre, University of Malawi College of Medicine, Private Bag 360, Chichiri, Blantyre, Malawi; Malaria Branch, Centers for Disease Control and Prevention (CDC), 1600 Clifton Road, Mail Stop A-06, Atlanta, GA 30333 USA

**Keywords:** Malaria, Febrile children, Long-lasting insecticide-treated nets, Prevention, Pyrethroid resistance

## Abstract

**Background:**

The escalating level of mosquito resistance to pyrethroid insecticides threatens the effectiveness of insecticide-treated nets (ITNs) for malaria control in Malawi. An evaluation of the effectiveness of ITNs for preventing malaria in children aged 6–59 months old, after 1 year of mass distribution of LLINs was conducted in Machinga District, Malawi, an area of moderate pyrethroid resistance.

**Methods:**

A facility-based, case–control study among children 6–59 months was conducted in an area of pyrethroid resistance between March and September 2013 in Machinga District. Cases and controls were children with fever who sought care from the same hospital and tested positive and negative, respectively, for malaria parasites by microscopy.

**Results:**

A high proportion of both cases (354 of 404 or 87.6 %) and controls (660 of 778 or 84.8 %) slept under an ITN the night before the survey. In univariable logistic regression, older age (24–59 months versus 6–23 months, p < 0.001), sleeping on the floor versus a mattress (p < 0.001), and open versus closed house eaves (p = 0.001) were associated with increased odds of malaria, whilst secondary education of the caretaker, having windows on multiple walls, and being in the least poor wealth quintile (p < 0.001 for each) reduced the odds of malaria; ITN use was not associated with malaria (p = 0.181). In multivariable analysis, older age (p < 0.001) and secondary education of the caregiver (p = 0.011) were the only factors significantly associated with malaria.

**Conclusion:**

This study did not find a significant personal protective effect of ITNs. However, high use of ITNs in the community and recent findings of lower malaria incidence in ITN users compared to bed net non-users from a cohort study in the same area suggest that ITNs provide community protection to both users and non-users alike in this area.

## Background

Insecticide-treated bed nets (ITNs) remain a cornerstone of global malaria control. Their excito-repellency effect deters mosquitoes from entering houses and causes premature exit from houses where ITNs are in use [1]. As a result, ITNs can reduce deaths and malaria morbidity [2] when used by individuals and when widely used they can provide community-wide protection even to people not sleeping under them by killing the mosquitoes and reducing vector abundance [3]. In the last few years, large-scale ITN distribution has increased in malaria-endemic areas. The number of nets delivered annually by manufacturers to countries in sub-Saharan Africa has increased from 6 in 2004 to 145 million in 2010, and ITN ownership has risen from 3 in 2000 to 54 % in 2013 [4]. The scale-up of ITNs and indoor residual spraying (IRS), and the widespread use of pyrethroids in agricultural pest management have contributed to the development of resistance to pyrethroids, the only class of insecticide that is approved for use on ITNs. The development and spread of pyrethroid resistance raises concerns about the effectiveness of ITNs as a malaria control intervention [5]. Although 27 countries in sub-Saharan Africa have reported pyrethroid resistance in *Anopheles* vectors [6], there is a dearth of epidemiological data on how resistance impacts the effectiveness of ITNs in reducing malaria infections. In Malawi, the escalating level of insecticide resistance to pyrethroids [7] led to the National Malaria Control Programme’s support of the use of pirimiphos-methyl (an organophosphate) to replace pyrethroids for IRS in selected districts in 2011. With ITNs however, no insecticide class besides pyrethroids is currently approved, and questions remain as to whether ITNs still provide protection against malaria in the face of intensifying pyrethroid resistance.

In this study, results are presented from a prospective clinic-based, case–control study conducted to evaluate the effectiveness of ITNs in preventing malaria amongst children aged 6–59 months in an area of moderate insecticide resistance to pyrethroids in Malawi.

## Methods

### Study setting

**Fig. 1:**
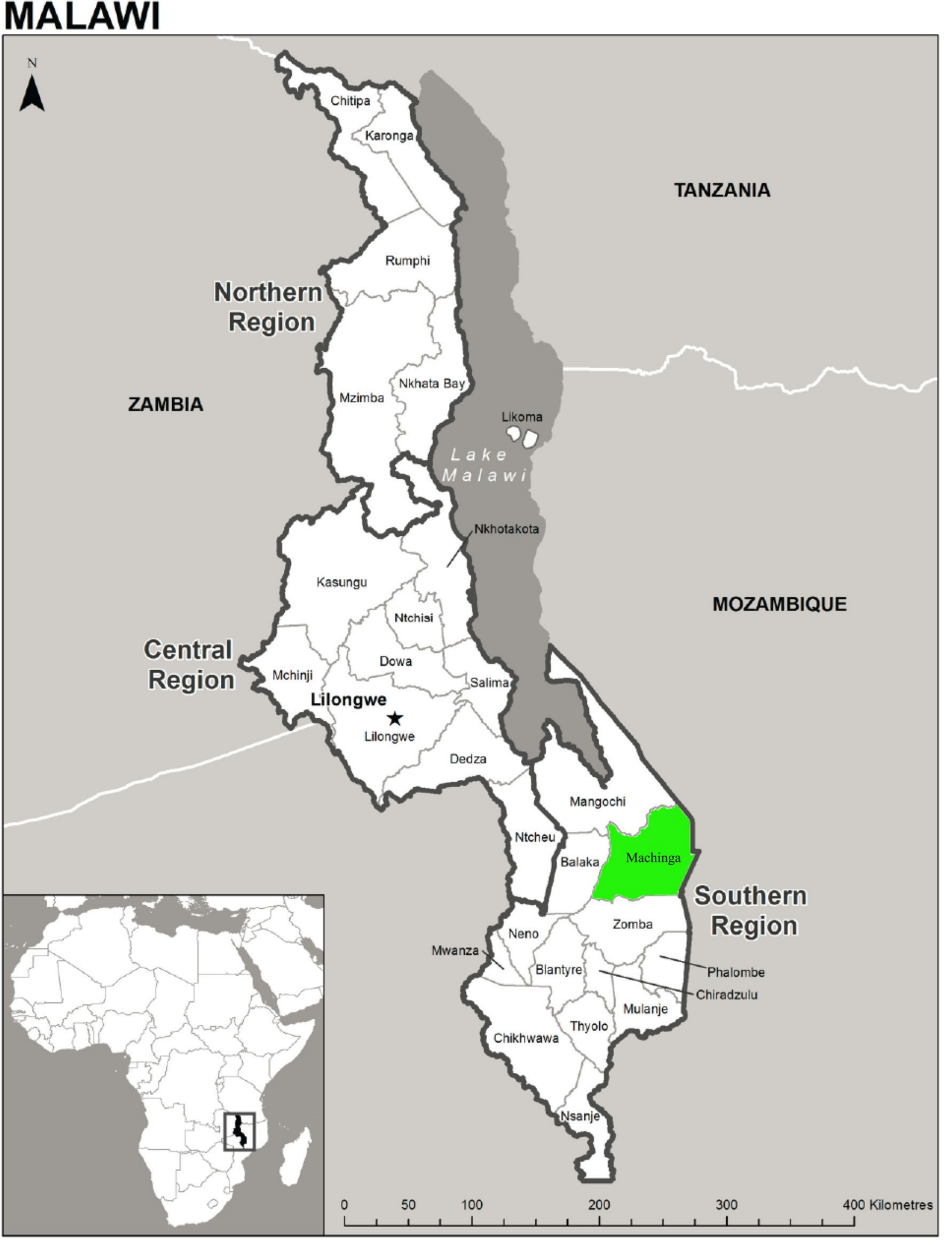
Map of Malawi showing study site

This study was conducted in the under-five outpatient clinic of Machinga District Hospital in southern Malawi (latitude −15°3′42.89, longitude 35°13′28.33), see Fig. 1. This government-run hospital provides primary health care for the population that surrounds it and secondary care for the entire district. In Machinga, malaria transmission is intense and year-round, peaking during the rainy season (November through May). *Plasmodium falciparum* is the dominant parasite species although *Plasmodium malariae* and *Plasmodium ovale* have been recorded [7]. *Anopheles funestus* is the main vector for malaria transmission in the area. Recent data on mortality of *An. funestus s.s.* exposed to pyrethroids show high levels (<50 % mortality) of pyrethroid resistance which is way below the 90 % cut-off point recommended by the WHO, in all districts where entomologic resistance monitoring has been carried out using the standard WHO test kits and procedures, including the study area [8]. Malaria control efforts in the study area are based on prompt diagnosis, a standard clinical guideline to test every patient for malaria with an RDT within 24–48 h of the onset of fever and treatment of cases with artemisinin-based combination therapy and on the use of ITNs. At the time of this study in 2013, a mass distribution campaign of Olyset^®^ Net (Sumitomo Chemical Co., Japan) ITNs had been completed in the district in July 2012, during which one ITN for every two people was distributed.

### Study design

This clinic-based, case–control study recruited cases and controls between March and September 2013. To be eligible to participate, children had to be between 6 and 59 months of age, living within 15 km of the hospital and not participating in another ongoing malaria study. Age-eligible children presenting at the Machinga District Hospital under-five outpatient department with current fever (measured axillary temperature ≥37.5 °C) or history of fever in the previous 48 h (as per caretaker) were invited to participate. After written informed consent was obtained from a caregiver, children were enrolled in the study. A questionnaire about illness history, ITN use, socio-economic status (SES) and other malaria risk factors was administered, and a thick and thin blood smear was collected from each participant. Rapid diagnostic tests for malaria (Paracheck Pf^®^ device, Orchid Biomedical Systems, India) were performed on all children and patients testing positive were treated by health facility staff. Data were collected using personal digital assistant (Dell Axim X51s, Dell Inc, Austin, TX, USA) with programmed skip patterns and logic checks.

Within 2 weeks of the clinic visit, surveyors that were employed and trained for 1 week on the study SOPs visited each participant at their home. Data on housing construction materials and environmental risk factors, such as standing water within 20 m of the sleeping structure, defined as the house where the child slept the night before the home visit, and visual inspection on the presence of nets, brand and condition of the nets were also collected.

### Laboratory analysis

Malaria parasites were detected through microscopy by staining thick and thin blood smears with 3 % Giemsa for 45 min. Blood smears were double-read read by study microscopists. Parasite density was calculated by counting the number of asexual parasites per 200 white blood cells, assuming a white blood cell count of 8000/μL of blood. Slides were considered negative if no parasites were found after examining fields containing at least 1000 white blood cells. Readings were considered to be discrepant if they differed by a factor of two or more for moderate or high-density parasitaemia slides (≥400 parasites/μL) or by a factor of 10 or more for low-density parasitaemia slide (<400 parasites/μL). Any discrepant readings were resolved by a third microscopist.

### Data analysis

Cases were defined as enrolled children with current measured temperature or recent reported fever (within 48 h) and a positive blood smear result for *P. falciparum* asexual stage parasites, once blood smears were read later on at the study laboratory. Controls were enrolled children with current or recent fever and a negative blood smear for *P. falciparum*. Data were analysed using STATA version 12 (College Station, TX, USA). A SES index was calculated using principal component analysis taking into account 14 household factors including: electricity, caregiver’s and spouse’s occupation, ownership of various assets, source of water, type of toilet, and material of the floor and roof [9]. The first principle component, which explained 25 % of the variance, was used to create an SES index and was used to divide the sample children into quintiles.

Univariable and multivariable logistic regression analyses were conducted to examine the relationship between potential risk factors and malaria. Only factors that were significant in univariable logistic regression analysis were included in a multivariable analysis using logistic regression. Odds ratios (ORs), adjusted odds ratios (AOR), and 95 % confidence intervals (CIs) were calculated in the analyses. For all statistical tests, a *P* value <0.05 was considered significant.

## Ethics

Written informed consent was obtained from parents or caregivers of each study participant prior to enrolment in the study. The study protocol was reviewed and approved by the institutional review boards of the University of Malawi, College of Medicine, Blantyre, Malawi and the Centers for Disease Control and Prevention, Atlanta, GA, USA.

## Results

A total of 403 cases and 778 controls had complete data and a household visit and were included in the final analysis. For 34 children (2.6 %), caregivers refused the home visit, and for 71 (5.5 %), homes were unable to be located after the clinic visit. There were no differences by sex between cases and controls. Median household size (IQR) was 4 (3–5) for both cases and controls. A high percentage of both cases (87.6 %) and controls (84.8 %) slept under an ITN the night prior to enrolment, and nearly as many were reported to have consistent net use, defined as sleeping under an ITN all 14 nights in the 2 weeks before illness onset (87.3 and 84.5 %, respectively) (Table 1). Among ITNs that children slept under, 42.0 % were reported to have any holes, and 13.7 % had holes that were fist-sized or larger (Table 1). Very few cases (5.9 %) or controls (4.6 %) slept in houses where a mosquito repellent was used the previous night. Approximately 20 % of children lived in homes with breeding sites present within 20 m, but this did not differ between cases and controls, p = 0.946.

**Table 1:** Demographic, socio-economic, and behavioral characteristics of the study participants presenting to Machinga District Hospital

Variable	Cases (n = 403) (%)	Controls (n = 778) (%)	Total	*p* value*
Age of child (months)				
6–23	188 (46.8)	515 (66.4)	703 (59.7)	
24–35	100 (24.9)	130 (16.8)	238 (19.5)	
36–59	114 (28.4)	131 (16.9)	245 (20.8)	<0.001
Sex				
Male	213 (53.0)	404 (52.0)	617 (52.4)	
Female	189 (47.0)	372 (47.9)	561 (47.6)	0.764
Slept under an ITN last night				
Yes	353 (87.6)	660 (84.8)	1009 (85.8)	0.199
No	50 (12.4)	118 (15.2)	168 (14.2)	
Slept under an ITN in 2 weeks before illness				
Yes	352 (87.3)	657 (84.5)	1009 (85.4)	0.181
No	51 (12.7)	131 (15.6)	168 (14.2)	
Physical integrity of child’s ITN				
Any holes	168 (44.3)	294 (40.8)	462 (42.0)	0.265
At least one fist-sized hole	57 (15.0)	93 (12.9)	150 (13.7)	0.330
Use of mosquito repellent				
Yes	24 (5.9)	36 (4.6)	60 (5.1)	0.326
No	379 (94.0)	742 (95.3)	1121 (94.9)	
Education of caretaker				
None	45 (11.2)	54 (7.0)	99 (8.4)	
Primary	308 (76.6)	549 (70.8)	857 (72.8)	
Secondary	49 (12.2)	172 (22.3)	222 (18.9)	<0.001
Child sleeps on				
Mattress	62 (16.4)	188 (26.2)	250 (22.8)	
Floor	316 (83.6)	529 (73.8)	845 (77.2)	<0.001
SES				
Least poor quintile	48 (11.9)	187 (23.0)	235 (20.0)	<0.001
Bottom 80 %	354 (88.1)	589 (75.9)	943 (80.1)	
Presence of 2+ windows not on same wall				
Yes	78 (26.7)	225 (37.6)	303 (34.0)	<0.001
No	205 (73.7)	354 (62.8)	559 (66.4)	
Eaves				
Any open	337 (83.6)	556 (75.3)	923 (78.2)	0.001
Closed	66 (16.4)	192 (24.7)	258 (21.9)	
Breeding sites within 20 meters				
Yes	82 (20.4)	157 (20.2)	239 (20.2)	0.946
No	321 (79.7)	621 (79.8)	942 (79.7)	

In univariable logistic regression analysis, neither sleeping under an ITN the previous night (OR 1.26, 95 % CI 0.88–1.80, p = 0.199) nor consistent net use immediately prior to the illness (OR 1.27, 95 % CI 0.89–1.81, p = 0.181), was associated with a reduction in the odds of malaria (Table 2). The results have shown an increased odds of malaria for older compared to younger children (6–23 months) (OR 2.11 for children 24–35 months, 95 % CI 1.55–2.87, p < 0.001, and OR 2.38 for children 36–59 months, 95 % CI 1.76–3.22, p < 0.001), for sleeping on the floor versus a mattress (OR 1.81, 95 % CI 1.32–2.49, p < 0.001), and for living in a house with open versus closed eaves (OR 1.67, 95 % CI 1.23–2.38, p = 0.001). Secondary education of the caretaker (OR 0.34, 95 % CI 0.20–0.56, p < 0.001), being in the least poor SES quintile (OR 0.43, 95 % CI 0.30–0.60, p < 0.001) and living in a house with at least one window on each of two or more different walls (OR 0.59, 95 % CI 0.44–0.79, p < 0.001) reduced the odds of malaria in univariable analysis (Table 2).

**Table 2:** Univariable and multivariable logistic regression analysis of various predictors and malaria

Variable	Univariable			Multivariable		
	Unadjusted OR	95 % CI	p value	Adjusted OR	95 % CI	p value
Age of child (months)						
6–23	REF			REF		
24–35	2.11	[1.55–2.87]	<0.001	2.23	[1.60–3.10]	<0.001
36–59	2.38	[1.76–3.22]	<0.001	2.84	[2.04–3.95]	<0.001
Slept under ITN 2 weeks before illness						
Yes	1.27	[0.89–1.81]	0.181			
No	REF			REF		
Slept under ITN last night						
Yes	1.26	[0.88–1.80]	0.199	1.08	[0.64–1.84]	0.765
No	REF			REF		
Education of caregiver						
None	REF			REF		
Primary	0.67	[0.44–1.02]	0.065	0.75	[0.48–1.17]	0.199
Secondary	0.34	[0.20–0.56]	<0.001	0.48	[0.27–0.84]	0.010
Child sleeps on						
Mattress	REF			REF		
Floor	1.81	[1.32–2.49]	<0.001	1.24	[0.86–1.79]	0.25
SES						
Least poor quintile	0.43	[0.31–0.60]	<0.001	0.65	[0.42–1.01]	0.054
Bottom 80 %	REF			REF		
Presence of 2+ windows not on same wall						
Yes	0.59	[0.44–0.79]	<0.001	0.72	[0.51–1.01]	0.054
No	REF			REF		
Eaves						
Any open	1.67	[1.23–2.28]	0.001	1.35	[0.91–1.99]	0.135
All closed	REF			REF		

After controlling for all variables significant in univariable analysis, only age 24–35 months (AOR 2.23, 95 % CI 1.60–3.10, p < 0.001), age 36–59 months (AOR 2.84, 95 % CI 2.04–3.95, p < 0.001) and having a caregiver with at least secondary education (AOR 0.48, 95 % CI 0.27–0.84, p = 0.010) remained statistically significantly associated with malaria (Table 2). Being in the least poor quintile (AOR 0.65, 95 % CI 0.42–1.01, p = 0.054) and having a window on at least two walls (AOR 0.72, 95 % CI 0.51–1.01, p = 0.054) were still associated with malaria but were only marginally statistically significant when adjusting for other factors. Sleeping under an ITN the previous night was not associated with malaria infection (AOR 1.08, 95 % CI 0.64–1.84, p = 0.765).

## Discussion

This case–control study did not show a personal protective effect of ITNs against malaria illness in children attending the outpatient clinic at Machinga District Hospital. The use of ITNs the night before the survey was high for both cases (87.6 %) and controls (84.8 %), which may be attributable to a national-wide mass ITN distribution campaign approximately 1 year before the study. The ITN distribution campaign was part of a universal coverage campaign that Malawi had in 2012, during which one ITN was distributed per two residents, with an extra net for households with an odd number of inhabitants. Thus, nearly all households in the study area owned at least one ITN during the study period. This lack of an individual-level protective effect could therefore be due to community-wide protective effects of ITNs in the study area. Research has shown that ITNs confer protection on community members who may not sleep under a net, even with modest ITN coverage of 35–65 % use among all ages, especially if the nets are of good quality [10]. The community-wide effect is achieved in areas where ITNs are widely used through the decreased abundance of indoor resting mosquitoes [11], feeding mosquitoes [12] larvae [13], reduced numbers of *Anopheles gambiae* and *An. funestus* in houses that are close to houses with an effective ITN [14] and reducing the average age of malaria vectors and therefore reducing the odds of a mosquito surviving long enough to transmit the parasite [15]. Even in areas of strong pyrethroid resistance, older more epidemiologically important mosquitoes are killed and thus a mass effect can still be achieved [16]. Improved health outcomes on child mortality, anaemia and parasitaemia have been demonstrated even for children not sleeping under an ITN if there is high ITN coverage in the entire population [3].

The finding of widespread resistance to *An. funestus* in this area [8] and elsewhere in Malawi [17] raises the possibility that insecticide resistance compromises the efficacy of ITNs, hence the lack of protective effects in this study. However, a recent systematic review and meta-analysis found that ITNs are still more effective in terms of entomological outcomes (e.g., mosquito mortality, knockdown, blood feeding, and induced exophily) than untreated bed nets even in areas with high resistance [6]. In addition, a recent cohort study in the same area found that the incidence of malaria infection in children using ITNs was significantly reduced compared to children who did not use ITNs [8]. These conflicting results could also be explained by the different methodologies used, especially considering the fact that case control studies face well described problems of bias and confounding [18]. Abdulla and others [19] showed that clinic-based, case–control studies may not be appropriate in the evaluation of an ITN programme because of attendance bias, where children with ITNs are more likely to visit the clinic when ill compared to those without ITNs. Since the data from community controls were not collected, it is difficult to assess the effect of attendance bias in this study [20] and it cannot be ruled out in explaining why this study did not show an individual protective effect of ITNs against malaria.

In addition, the lack of protective [21] efficacy could be due to decline in the physical integrity and biological activity of insecticides on these ITNs, a problem which has been highlighted in the region [22]. The efficacy of ITNs can only be guaranteed if nets are not worn out and have not lost the potency of the insecticide. Although this study was conducted only a year after a mass distribution campaign, almost half of the nets amongst both cases and controls had already developed holes, and the use of nets was through caregiver report. In addition, insecticide levels on the nets were not measured in this study.

This study indicated that caregiver’s education was associated with lower risk of malaria, as was living in a higher SES household and having windows on at least two different walls, although the latter two were only marginally statistically significant when adjusted for other factors. This is consistent with findings from a recent meta-analysis which showed that the odds of malaria were higher in the poorest compared to the least poor children [23] and recent research showing a strong relationship between maternal education and reduced risk of malaria infection [23]. This study also showed that housing characteristics such as closed eaves and the presence of windows on at least two walls of the house were associated with reduced risk of malaria. These findings are supported by several other studies within the region which have shown that the risk of malaria transmission is increased in households with open eaves and without windows [24]. In this study, the majority of houses occupied by cases (83.6 %) and controls (75.3 %) had open eaves and very few houses had more than two windows. This highlights that simple interventions such as good housing construction could go a long way in preventing malaria in this community [25]. However, adjusted for other factors, including caregiver’s education and SES, housing characteristics were no longer significant protective factors against malaria, which could be explained by the fact that wealthier households tend to have better housing conditions.

## Conclusion

The study did not find a significant personal protective effect of ITNs in an area of high ITN use and pyrethroid insecticide resistance. The lack of an individual protective effect could be due to overriding community-wide protection from ITNs in this area or limitations in the case–control study design. The use of ITNs should still be continued in the area because of their physical barrier that they provide and their community-wide effect by killing older more epidemiologically important vectors.
